# Silicon Modulates the Chloroplast Proteome to Enhance Drought Tolerance in Soybean

**DOI:** 10.3390/plants15030497

**Published:** 2026-02-05

**Authors:** Amandeep Kaur, Saroj Kumar Sah, Kambham Raja Reddy, Jiaxu Li

**Affiliations:** 1 Department of Biochemistry, Nutrition, and Health Promotion, Mississippi State University, Mississippi State, MS 39762, USA; 2 Department of Plant and Soil Sciences, Mississippi State University, Mississippi State, MS 39762, USA

**Keywords:** drought stress, silicon supplementation, soybean, chloroplast proteomics, photosynthesis, stress tolerance

## Abstract

Soybeans are highly susceptible to drought stress, which significantly impairs their growth and yield. Silicon (Si) supplementation has emerged as a promising strategy to mitigate drought-induced damage in plants. We investigated changes in the physiological and chloroplast proteomes in soybeans under drought stress, both with and without Si supplementation. Soybean plants were grown under controlled conditions and subjected to drought stress. The treatments included Si application (sodium silicate), sodium chloride control, and water control. Chloroplast proteins were extracted from control and Si-treated plants and analyzed using two-dimensional gel electrophoresis and mass spectrometry. Plants treated with Si showed improved drought tolerance, exhibiting reduced leaf rolling and wilting, while the control plants experienced significant wilting under drought conditions. Photosynthetic performance, measured by quantum efficiency of photosystem II and chlorophyll content, was better maintained in Si-supplemented plants under drought. However, stomatal conductance and transpiration were similarly reduced across all drought treatments. We detected 15 Si-responsive protein spots corresponding to 13 unique chloroplast proteins that were differentially expressed in response to Si supplementation. These identified proteins include those involved in photosynthesis, such as Rubisco activase isoforms, oxygen-evolving enhancer proteins, and PsbP domain-containing protein, as well as stress response proteins like dehydrin and 20 kDa chaperonin. Si treatment upregulated Rubisco activase isoforms, oxygen-evolving enhancer proteins, PsbP domain-containing protein, and 20 kDa chaperonin, which are typically reduced under drought. Si treatment maintained a higher glutamine synthetase level under drought stress. Gene ontology and KEGG pathway analyses revealed that Si-modulated proteins are associated with photosynthesis, energy metabolism, and nitrogen metabolism under drought stress. Our findings demonstrate that Si supplementation alleviates drought stress in soybean by preserving chloroplast function and enhancing the expression of photosynthetic proteins and enzymes, as well as key stress-responsive proteins. This research provides insights into the molecular mechanisms of Si-induced drought tolerance in soybeans and highlights potential targets for developing drought-resilient soybean cultivars.

## 1. Introduction

Soybeans are an important economic crop globally, valued for their high protein content, dietary minerals, and vitamins. It is a significant source of protein and oil in both the feed and food industries, serving as a good meat substitute for vegetarian diets [1]. In addition to its nutritional benefits, soybeans enhance soil fertility through nitrogen fixation [2] and provide raw materials for various industrial products, including vegetable oils, wax, paints, dyes, and fibers [3]. According to the United States Department of Agriculture, the U.S. is the world’s largest producer and a leading exporter of soybeans, highlighting the economic importance of maximizing soybean yields. To meet future demands, it will be necessary to develop strategies to improve soybean productivity, especially in challenging conditions.

Drought poses a significant threat to soybean production, as it requires substantial water for growth and reproduction [4]. Global warming and climate change are increasing the frequency of droughts, particularly in areas that rely on rainfed or limited irrigation [5,6]. Under drought conditions, soybean yields can decrease by more than 50%, resulting in considerable financial losses [7]. Typically, soybeans need between 450 and 700 mm of water throughout the growing season [8]. Drought during critical growth stages, such as flowering, pod set, and seed filling, can drastically reduce yield potential [9,10,11]. For instance, drought stress during the pod-filling stage has been reported to significantly lower pod and seed numbers per plant, which are essential components for yield [12,13]. In Mississippi, most soybeans are cultivated in dryland (non-irrigated) fields, making them especially vulnerable to drought-related yield losses. Therefore, effective mitigation strategies are necessary to sustain soybean yield under conditions of limited irrigation or rainfed conditions.

Silicon (Si) is increasingly recognized as a beneficial element in plant nutrition [14]. In the soil, the total silicon content may be large, but the amount of soluble silicon (silicic acid) available for plant uptake and utilization is limited [15]. Plants take up soluble silicon into their tissues, but it is not returned to the soil through biodegradation. Thus, continuous cropping of land can cause a deficiency of soluble silicon in the soil. Recent studies have shown that supplying crops with soluble silicon can enhance abiotic stress tolerance and thus increase crop productivity under adverse stress conditions [16,17]. Potential physiological mechanisms behind increased drought tolerance from silicon application include reduced water loss through transpiration at leaf stomata and the cuticle and increased water-use efficiency [16,17]. Another possible mechanism is that silicon can trigger metabolic changes, such as altering primary metabolism, stimulating photosynthetic rate, and increasing antioxidant enzyme activities [14].

Because drought-driven yield losses in soybean are tightly linked to declines in photosynthetic performance and increases in oxidative stress, the reported whole-plant benefits of silicon (Si) under water deficit (e.g., improved water-use efficiency, sustained photosynthetic capacity, and enhanced antioxidant activity) naturally point to the chloroplast as a key mechanistic site. Chloroplasts are not only the primary site of photosynthesis but also major hubs for stress signaling and ROS generation/detoxification; therefore, modest shifts in chloroplast-localized protein networks can translate into measurable improvements at the leaf and whole-plant levels [18]. Despite this logic, how Si reshapes the chloroplast protein landscape during drought remains insufficiently defined, particularly in soybean, an intermediate Si accumulator [19]. Consistent with the central role of chloroplast homeostasis in stress acclimation, prior work has highlighted the sensitivity of chloroplast function to abiotic perturbations and the importance of chloroplast-localized protective systems (including heat shock proteins/chaperones) in sustaining growth under stress [20,21]. Emerging evidence also suggests that Si can help preserve chloroplast integrity and the photosynthetic apparatus during drought by limiting chlorophyll degradation, maintaining chlorophyll fluorescence traits, and supporting stress-protective proteins across species [22,23,24]. Building on this framework, we used chloroplast-targeted proteomics to connect Si-associated whole-plant physiological benefits with chloroplast protein-level responses under drought stress in soybean.

## 2. Results

### 2.1. Effect of Silicon on Plant Growth and Drought Tolerance

**Figure 1:**
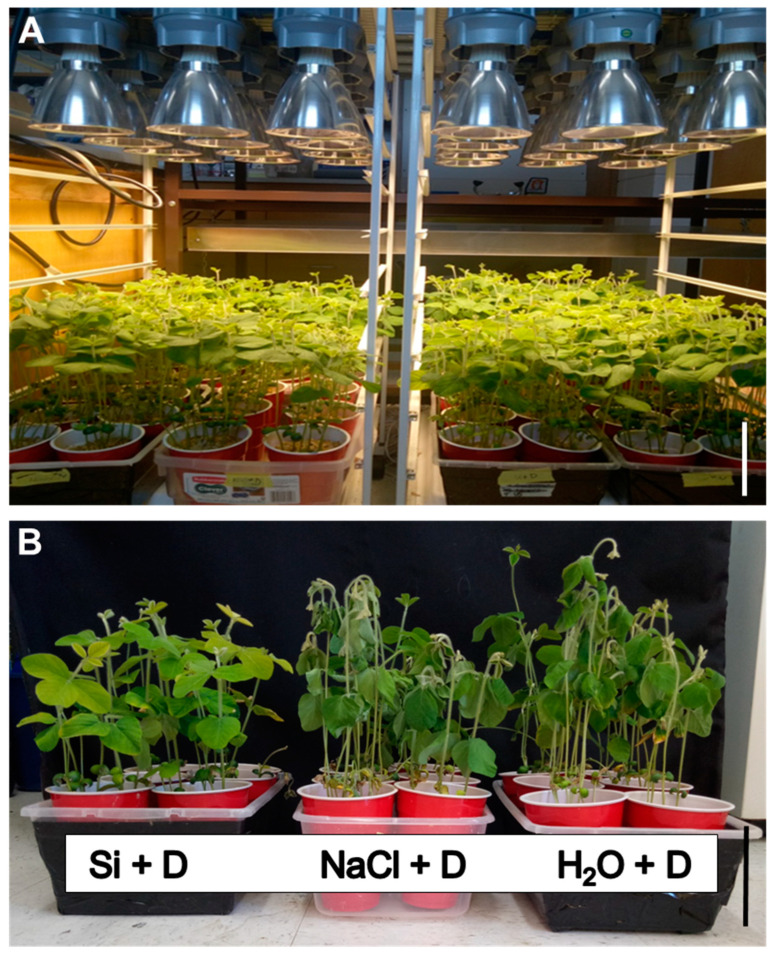
Effects of silicon supplementation on soybean drought tolerance. Soybeans were grown in cups containing sand supplied with the same volume of distilled water (H_2_O), 2 mM Na_2_SiO_3_ (Si), or 4 mM NaCl solution. The 4 mM NaCl solution was used to balance the same total sodium in the Na_2_SiO_3_ solution so as to identify only the effect of silicate. Drought (D) was imposed by withholding irrigation for 7 days at the second trifoliate stage. (**A**) Soybean plants before drought imposition. (**B**) Soybean plants after 7 days of drought. Scale bar = 10 cm.

Before drought imposition, all soybean plants grew uniformly under well-watered conditions (Figure 1A). However, after 7 days without watering, significant differences were observed among the treatments. Plants in the water control and NaCl control groups (no Si) exhibited noticeable leaf wilting and rolling under drought stress. In contrast, plants supplemented with Si (Na_2_SiO_3_) maintained an upright, turgid appearance, like well-watered controls. The Si-treated plants survived the 7-day drought without severe wilting (Figure 1B), while many leaves of non-Si-treated plants became desiccated. These phenotypic observations suggest that Si application significantly improved soybean drought tolerance by helping maintain leaf turgor and improving plant survival.

### 2.2. Effect of Silicon on Photosynthetic Parameters and Pigment Contents

**Figure 2:**
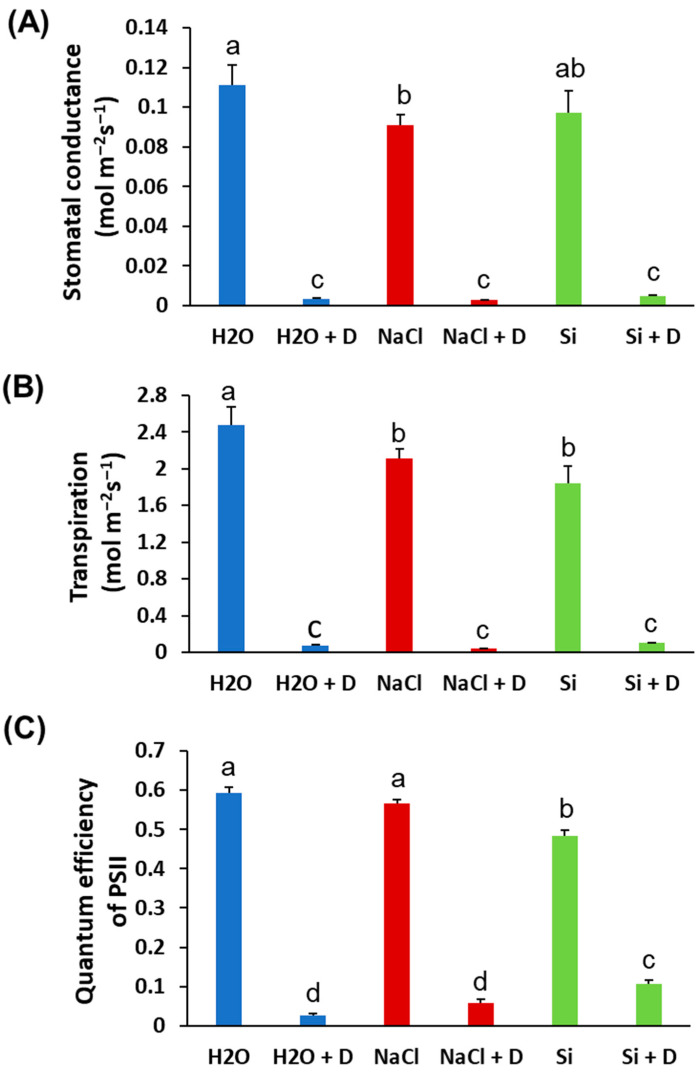
Physiological parameters of soybean plants subjected to water deficit stress. Soybeans were grown in cups containing sand supplied with the same volume of distilled water (H_2_O), 2 mM Na_2_SiO_3_ (Si), or 4 mM NaCl solution. Hoagland nutrient solution was supplied to all plants to provide baseline nutrition. These plants were then subjected to drought (D) treatment by stopping irrigation. (**A**) Stomatal conductance, (**B**) transpiration, and (**C**) quantum efficiency of PSII. Different letters denote statistically significant differences (*p* < 0.05, Tukey’s test). Values represent means ± SE (n = 3).

**Figure 3:**
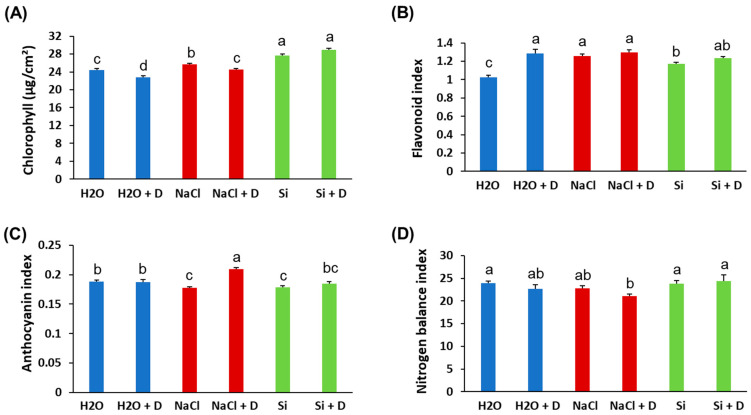
Leaf pigment alterations of soybean plants subjected to water deficit stress. Soybeans were grown in cups containing sand supplied with the same volume of distilled water (H_2_O), 2 mM Na_2_SiO_3_ (Si), or 4 mM NaCl solution. Hoagland nutrient solution was supplied to all plants to provide baseline nutrition. These plants were then subjected to drought (D) treatment by stopping irrigation. (**A**) Chlorophyll content, (**B**) flavonoid index, (**C**) anthocyanin index, and (**D**) nitrogen balance index. Different letters denote statistically significant differences (*p* < 0.05, Tukey’s test). Values represent means ± SE (n = 3).

Baseline (pre-drought) physiological measurements did not differ significantly among treatments; therefore, we present end-point values at the end of the 7-day drought period. Stomatal conductance and transpiration rates declined in all plants under drought, as expected, and Si treatment did not prevent these reductions. There were no statistically significant differences in stomatal conductance or transpiration between Si-treated and control plants under either well-watered or drought conditions (Figure 2A,B). Under drought stress, the quantum efficiency of photosystem II (ΦPSII) decreased in control (H_2_O) and NaCl-treated plants relative to their well-watered counterparts. However, Si-treated plants under drought retained higher ΦPSII than the drought-stressed controls. Specifically, ΦPSII in Si + D plants was significantly greater than in control drought-stressed plants (*p* < 0.05), approaching the levels of well-watered plants (Figure 2C). This indicates that Si helped preserve the photochemical efficiency of PSII during drought. Consistent with the ΦPSII results, total chlorophyll content was better maintained in Si-treated plants under drought. Drought stress reduced chlorophyll levels in control and NaCl-treated plants. However, the plants treated with Si under drought conditions exhibited chlorophyll levels even higher than those of the unstressed controls (Figure 3A). Chlorophyll content was highest in the Si-treated group under both watered and drought conditions, suggesting a positive effect of Si on pigment content irrespective of moisture status. In contrast, leaf flavonoid and anthocyanin levels increased significantly under drought in control and NaCl-treated plants (as typical stress-induced secondary metabolites), but these increases were mitigated by Si, which mitigated these increases. Si-treated drought-stressed plants accumulated much lower levels of flavonoids and anthocyanins compared to drought-stressed controls (Figure 3B,C). The flavonoid and anthocyanin indices in Si + D plants were comparable to those in non-stressed plants, indicating that Si reduced the need for these stress-related compounds, likely by alleviating oxidative stress. Meanwhile, the nitrogen balance index, the ratio of chlorophyll to flavonoids, did not differ significantly among treatments (Figure 3D), implying that overall nitrogen status was similar. These physiological measurements demonstrate that Si supplementation helped maintain photosynthetic function (higher chlorophyll, higher ΦPSII) and reduced stress-induced pigment changes under drought. Because Si maintained photosynthetic performance under drought (higher ΦPSII and chlorophyll) even though stomatal conductance and transpiration declined similarly across drought treatments, we next tested whether Si alters drought-responsive pathways at the chloroplast protein level using chloroplast-targeted proteomics.

### 2.3. Differentially Expressed Chloroplast Proteins in Si-Treated Plants

**Figure 4:**
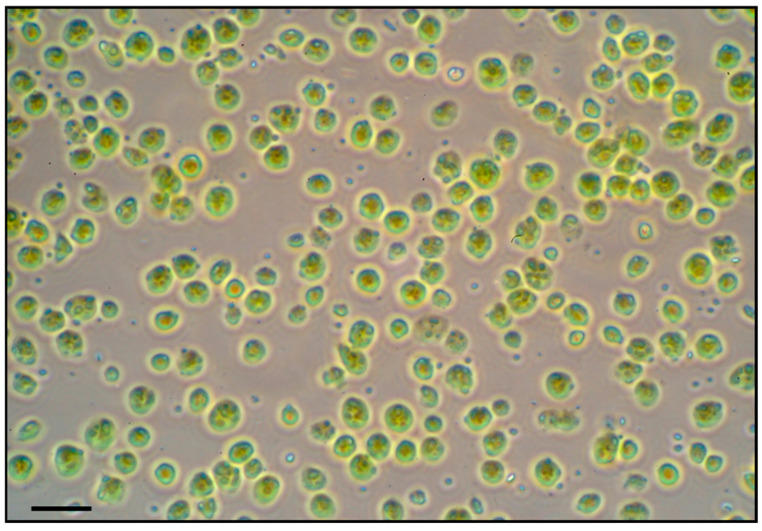
Light microscopic image of intact chloroplasts isolated from soybean leaves. Intact chloroplasts were isolated by Percoll density gradient centrifugation. Isolated soybean chloroplasts were viewed by brightfield microscopy. Scale bar = 10 µm.

Intact chloroplasts were successfully isolated from soybean leaves of all treatments following the Percoll gradient method. The yield of intact chloroplasts was sufficient (approximately 2 mL of concentrated chloroplast suspension per 10 g of leaf tissue) for two-dimensional gel electrophoresis (2-DE)-based proteomic analysis. The microscopy of isolated chloroplasts confirmed that a high proportion of chloroplasts were intact and morphologically normal in all samples (Figure 4). We did not observe any obvious structural differences in chloroplasts between treatments at the light microscopy level. Thus, any proteomic differences between treatments can be attributed to treatment effects rather than isolation artifacts.

**Figure 5:**
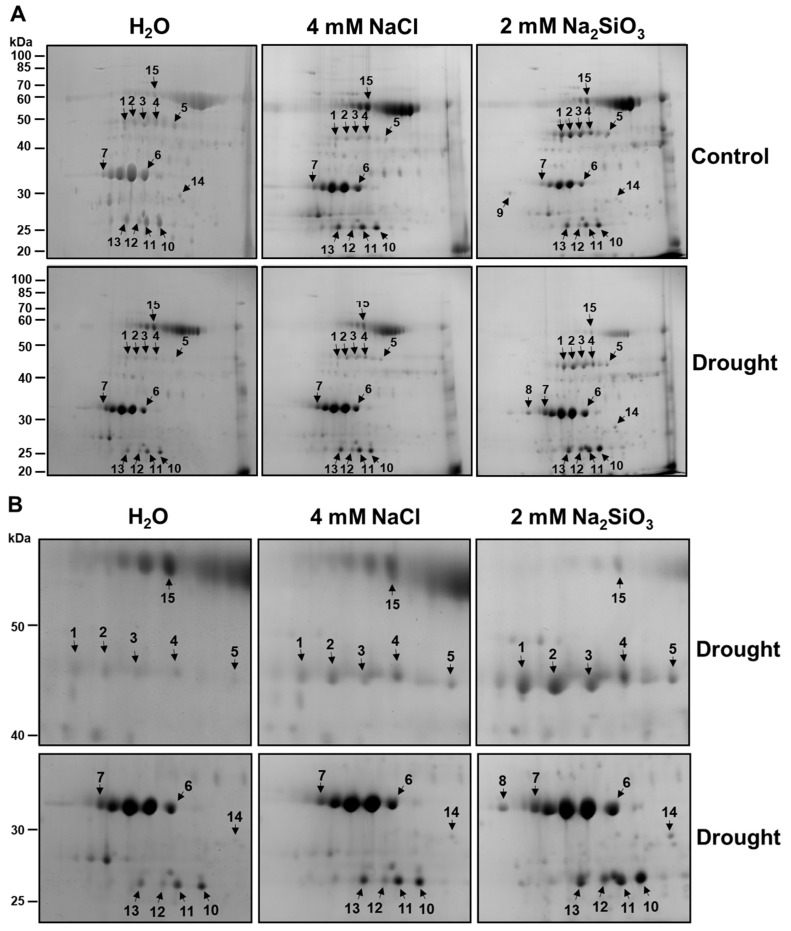
Two-dimensional gel analysis of chloroplast proteins of soybean plants in response to silicate application. (**A**) Soybeans were grown in cups containing sand supplied with the same volume of distilled water (H_2_O), 2 mM Na_2_SiO_3_ (Si), or 4 mM NaCl solution. These plants were then subjected to drought treatment by stopping irrigation. Chloroplast proteins (500 µg) separated by two-dimensional gel electrophoresis were stained with colloidal Coomassie blue. Silicate-responsive proteins were indicated with arrows. The positions of molecular mass markers are shown in kilodaltons (kDa) at the left-hand margin. (**B**) Zoomed-in sections of silicate-responsive proteins under drought conditions.

Chloroplast protein extracts from drought-stressed and well-watered plants (with or without Si treatment) were separated by 2-DE (two-dimensional gel electrophoresis) to profile changes in the chloroplast proteome. Consistent, high-resolution 2-DE protein maps were obtained for each treatment (replicated gels, n = 3). The Coomassie-stained 2-DE gels showed approximately 100 distinct protein spots in the pI 4–7 range and molecular mass 15–100 kDa (Figure 5). Visual comparison of gels revealed a set of protein spots whose intensity differed between Si-treated and control plants under drought. We detected 15 protein spots that were either up-regulated or down-regulated in response to Si treatment. These spots were numbered 1–15 on the gels (Figure 5, arrows). All 15 spots were excised, digested with trypsin, and analyzed by tandem mass spectrometry.

The identities and properties of these differentially expressed proteins are summarized in Table 1. Table 1 also provides each protein’s UniProt accession, gene locus, theoretical molecular weight (MW) and isoelectric point (pI), and experimental MW and pI of proteins. The 15 protein spots (Figure 5) were identified by mass spectrometry and matched to 13 unique proteins (Table 1), as two proteins appeared as two different spots (spots 1 and 2: Rubisco activase isoform C; spots 3 and 4: Rubisco activase isoform A). The same protein can appear as multiple spots on a 2-DE gel because post-translational modifications such as phosphorylation and acetylation alter its isoelectric point, causing the protein with modifications and the non-modified protein to migrate differently on a 2-DE gel [25,26,27]. Overall, the experimentally derived MWs of the proteins matched the predicted theoretical MWs (Table 1). The experimentally derived pI values of most proteins were slightly lower than the predicted theoretical pIs, while three proteins (spot 13, 14, 15) showed higher experimental pI values than their theoretical pIs. Protein modifications that are adding negative charges (phosphorylation) or removing positive charges (acetylation) can lower proteins’ pI, whereas protein modifications that are adding positive charges or removing negative charges (esterification) can increase proteins’ pI values [26].

Notably, many of the identified proteins are directly involved in photosynthesis, stress response, or chloroplast RNA processing, suggesting that drought and Si treatments induce specific adjustments in the chloroplast proteome. In general, drought stress in control plants decreased in photosynthesis-related proteins. In contrast, Si-treated plants under drought maintained or increased levels of these proteins (Table 1 and Supplementary Table S1). Detailed alterations in protein abundance in response to Si and drought treatments are described below.

**Rubisco activase (RCA):** Spots 1 and 2 were identified by mass spectrometry as the chloroplast isoform of Rubisco activase C (RCA β), and spots 3 and 4 as Rubisco activase A (RCA α) (Figure 5, Table 1). Drought-stressed control plants showed markedly lower levels of both RCA α and RCA β spots compared to well-watered controls (spots 1–4 were faint in H_2_O + Drought gel in Figure 5). In contrast, Si-treated plants under drought maintained high levels of RCA α and RCA β, comparable to those in well-watered conditions (Figure 5). In fact, RCA spots were among the most strongly enhanced by Si under drought. This suggests that Si helped sustain Rubisco activation capacity during drought. These results align with previous findings that reduced RCA leads to reduced photosynthesis under stress and that maintaining RCA can improve stress tolerance [28].

**Oxygen-evolving enhancer proteins (OEE):** Spots 6 and 7 were identified as oxygen-evolving enhancer protein 1 and 33 kDa subunit of oxygen-evolving system of photosystem II (OE33), respectively, while spots 10 and 11 were identified as oxygen-evolving enhancer protein 2 and oxygen-evolving enhancer protein 2-1, respectively. Oxygen-evolving enhancer protein 1 (OEE1) and oxygen-evolving enhancer protein 2 (OEE2) stabilize the oxygen-evolving complex of PSII and are known to be drought-sensitive proteins. We observed that drought-stressed control soybeans had significantly reduced levels of OEE1, OE33, and OEE2 spots (6, 7, and 10, 11) on 2-DE gels. In contrast, Si-treated plants under drought retained much higher levels of both proteins (Figure 5). In fact, OEE1, OE33, and OEE2 in Si and drought-treated plants were similar to those in non-stressed plants, while in control plants under drought, these spots were barely detectable (Figure 5). This implies that Si protected the PSII oxygen-evolving complex from drought-induced damage or degradation. Preserving OEE proteins in Si-treated plants would help maintain PSII function and support continued photosynthetic electron transport under drought. This agrees with the higher PSII efficiency measured in Si-treated plants (Figure 2). It is noteworthy that previous studies on beans found that genotypes-tolerant genotypes maintained OEE levels better than sensitive ones. Our results demonstrate that exogenous Si can induce a similar protective effect on these sensitive PSII components.

**PsbP domain-containing protein 6:** Spot 13 was identified as PsbP domain-containing protein 6 (PPD6). PsbP-domain proteins are auxiliary proteins in the PSII complex and have been implicated in stress responses. PPD6 protein level (spot 13, Figure 5) was increased in the Si-treated plants under drought compared to control plants under drought (Figure 5). This indicates Si induced or retained PPD6 during drought. Tamburino et al. [29] likewise found that PPD6 was induced in tomato chloroplasts during drought and recovery. Further research is needed to clarify the function of PPD6, but our results indicate that it is a Si-responsive protein under drought.

**Glutamine synthetase:** Spot 5 was identified as chloroplastic glutamine synthetase (GS2). The GS2 spot intensity was slightly reduced by drought in control plants. Still, notably, GS2 was more abundant in Si-treated plants under drought than in drought controls (spot 5, Figure 5). This suggests Si helped maintain GS2 levels under drought. Indeed, measurements of leaf NBI (nitrogen balance index) showed no drop in Si-treated plants (Figure 3), consistent with sustained nitrogen assimilation. Drought can disrupt nitrogen metabolism, and decreases in GS activity under drought have been reported in sensitive wheat and rice genotypes [30]. By supporting the GS function, Si could help preserve amino acid and protein synthesis under drought. This is in line with observations that transgenic approaches to enhance GS can improve plant growth under stress [31]. Our data imply that Si may alleviate drought’s impact on nitrogen metabolism by maintaining GS2 activity, thereby contributing to overall plant vigor under stress.

**ATP synthase β subunit:** Spot 15 was identified as the chloroplastic ATP synthase β subunit. ATP synthase β subunit (spot 15, Figure 5) was downregulated in Si-treated plants under drought compared to control plants under drought. The previous study shows chloroplastic ATP synthase content generally declines under drought, which can limit ATP availability for carbon fixation and other processes [32]. Under severe drought, a reduction in ATP synthase is expected as a protective measure to avoid excess ATP when demand is low [32]. Thus, maintaining a balance in energy production, rather than fully preventing the decline in ATP synthase, could explain why the benefit of Si is more pronounced for upstream components (RCA, OEE) than for ATP synthase itself.

**Splicing factor SF3b4 and RNA-binding protein:** Spot 8 was identified as splicing factor 3b subunit 4, a nuclear-encoded chloroplast RNA splicing factor. Spot 9 was an RNA-binding protein in chloroplasts. SF3b4 was only detected in Si-treated plants under drought (Figure 5). Similarly, a chloroplast RNA-binding protein was uniquely present in Si-treated well-watered plants (Figure 5). SF3b4 and the chloroplast RNA-binding protein may be involved in RNA processing and stability. These proteins might not directly contribute to drought tolerance but could be indicative of altered gene expression dynamics under Si treatment. Ambrosone et al. [33] noted that RNA-binding proteins can be key regulators of stress responses. The presence of specific RNA-binding proteins in Si-treated plants might reflect a Si-induced priming effect, where gene expression modulation helps the plant better cope with stress.

**Dehydrin and chaperonin:** Dehydrins and dehydrin-like proteins are present in the cytosol, chloroplasts, mitochondria, and nucleus, acting as protective chaperones against abiotic stress by stabilizing membranes and proteins [34]. We identified a dehydrin-like protein (spot 14) from chloroplasts that was more abundant in Si-treated plants under drought compared to control plants under drought (Figure 5). In drought-tolerant soybean varieties, higher dehydrin accumulation under drought is correlated with better performance [35]. Our results indicate that Si might increase protective dehydrin-like protein accumulation. Similarly, we identified a chloroplastic 20 kDa chaperonin (spot 12) was up-regulated by drought in Si-treated plants but showed less abundance in control plants under drought (Figure 5). CPN20 (20 kDa chaperonin) assists protein folding in chloroplasts and can support the function of antioxidant enzymes [36]. Our Si-treated soybeans showed a notable increase in CPN20 under drought (Figure 5), suggesting that Si actively promotes the protein-folding machinery in stressed chloroplasts, potentially preventing misfolding and aggregation of proteins during drought. The increased protein abundance of both dehydrin and CPN20 in Si-treated plants highlights Si’s role in bolstering protective stress responses at the protein level, which likely contributes to the improved physiological outcomes observed (Figure 1).

In summary, the chloroplast proteomic analysis revealed that Si supplementation under drought broadly counteracts the drought-induced loss of crucial photosynthetic and stress-related proteins. SI-treated drought-stressed soybeans maintained higher levels of Rubisco activases, OEE1, OEE2, OE33, PsbP-domain protein 6, glutamine synthetase, and protective proteins like dehydrin and chaperonin, compared to non-supplemented drought-stressed plants. These proteomic adjustments help explain the physiological improvements (better photosystem II efficiency, higher chlorophyll, less oxidative pigment accumulation) conferred by Si under drought (Figure 2 and Figure 3).

### 2.4. Functional Classification of Si-Responsive Proteins

**Figure 6:**
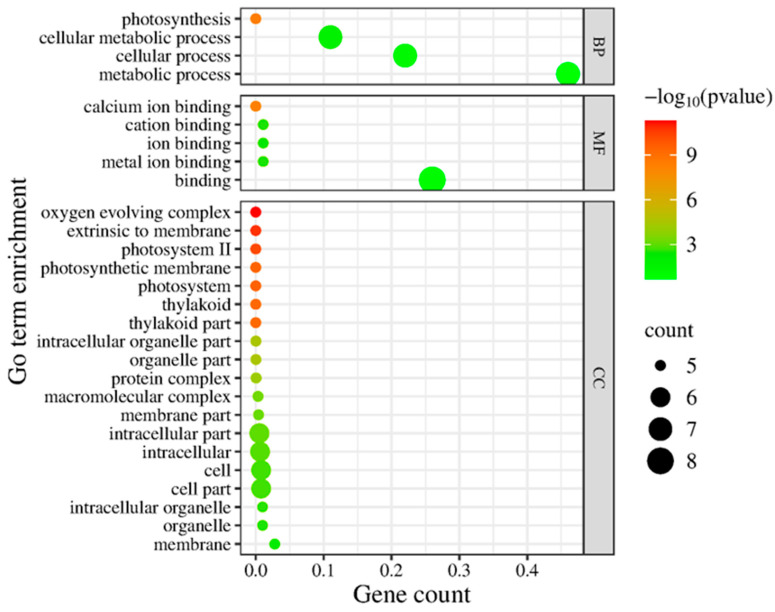
Gene Ontology (GO) enrichment analysis of differentially expressed chloroplast proteins. Enrichment of GO terms in biological process (BP), molecular function (MF), and cellular component (CC) was analyzed using AgriGO, and the graph was plotted using ggplot2. The significant terms (adjusted *p* < 0.05) are shown with colors.

**Figure 7:**
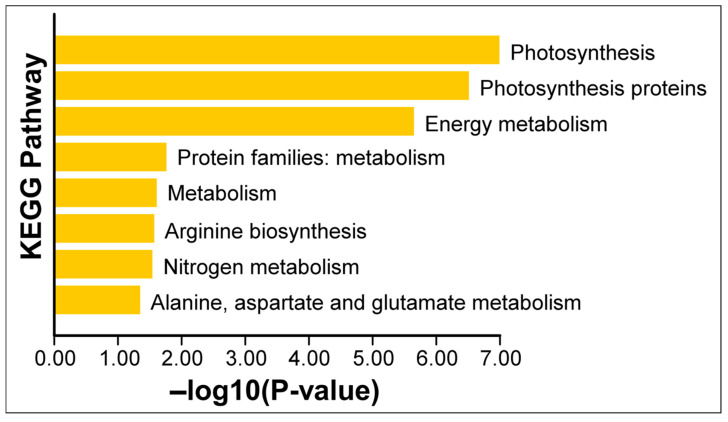
KEGG enrichment analysis of differentially expressed chloroplast proteins. Enrichment of the KEGG pathway was analyzed using Tbtools software (v1.116). The *p*-values of the KEGG enrichment terms shown here are <0.05.

To gain an overview of the functional implications of these proteomic changes, we performed GO (Gene Ontology) enrichment analysis on the 13 Si-responsive proteins. The GO biological process category confirmed that many of the Si-responsive proteins are involved in photosynthesis and cellular metabolic processes (Figure 6). For the molecular function category, the GO term for calcium ion binding or cation binding was enriched, reflecting the calcium-binding property of the identified oxygen-evolving enhancer proteins. In the cellular component category, the enriched GO terms include oxygen-evolving complex, photosystem II, photosynthetic membrane, and thylakoid (Figure 6). Next, we mapped the proteins to metabolic pathways using KEGG (Kyoto Encyclopedia of Genes and Genomes) enrichment analysis. Figure 7 illustrates a classification of the identified proteins in major KEGG pathways. The enriched KEGG pathways include photosynthesis, energy metabolism, arginine biosynthesis, and nitrogen metabolism (alanine, aspartate, and glutamate metabolism). Functional classification of the 13 Si-responsive proteins is summarized in Table 2. Seven of the proteins (Rubisco activase isoform A and C, oxygen-evolving enhancer proteins, PSBP domain protein 6) were associated with photosynthesis. Glutamine synthetase is a key enzyme in ammonia assimilation, linking nitrogen and carbon metabolism. Glutamine synthetase also appears in carbohydrate metabolism pathways due to its role in nitrogen re-assimilation during photorespiration. Other identified proteins did not map directly to specific KEGG pathways but are known to play roles in assisting protein folding (chaperonin), stress tolerance (dehydrin), or RNA processing and stability (RNA-binding protein, splicing factor). The pathway analysis supports the idea that Si’s effect under drought involves maintaining components of energy metabolism (photosynthesis) and nitrogen assimilation.

## 3. Discussion

### 3.1. Chloroplast-Level Perspective on Silicon Responses Under Drought

Silicon (Si) is increasingly recognized as a beneficial element that can improve plant performance under abiotic stresses. Previous work across cereals and dicots has largely emphasized whole-plant outcomes (water status, antioxidant capacity, and growth) but has provided limited resolution on how Si intersects with the chloroplast machinery that ultimately determines photosynthetic resilience under water deficit [49,50,51,52,53,54,55]. This knowledge gap is particularly relevant for soybeans, a dicot and intermediate Si accumulator, where Si-associated benefits may be subtle and strongly compartment-specific.

Here, we coupled physiological phenotyping with chloroplast-enriched proteomics to connect whole-plant benefits to subcellular protein networks. Prior chloroplast-focused proteomic studies of Si have been relatively rare and have mainly examined other stresses or species (e.g., tomato chloroplasts under salinity [54]); here we extend organelle-resolved proteomics to drought in soybean, a legume with intermediate Si accumulation. This approach is, to our knowledge, among the first to profile the soybean chloroplast proteome under drought with and without Si supplementation, and it identifies photosynthesis- and metabolism-related pathways (including photosynthesis proteins and nitrogen/central amino acid metabolism) as prominent Si-responsive nodes under drought (Figure 6 and Figure 7).

### 3.2. Silicon Improving PSII Performance Under Drought

Si-treated soybean plants displayed less wilting/leaf rolling during a short-term severe drought (Figure 1). Notably, drought reduced stomatal conductance and transpiration across treatments (Figure 2A,B), indicating that Si does not simply prevent stomatal closure under water deficit. Instead, Si-treated plants maintained significantly higher PSII quantum efficiency (ΦPSII) under drought (Figure 2C), consistent with protection of chloroplast photochemistry against drought-associated pho-toinhibition and oxidative damage.

### 3.3. Silicon Stabilizing Photosynthetic Proteins and Enzymes Under Drought

The proteomic data provide mechanistic support for this physiological pattern: drought alone decreased the abundance of multiple chloroplast proteins central to photo-synthetic performance, whereas Si supplementation prevented or attenuated many of these drought-driven reductions. In particular, Si maintained or increased proteins associated with PSII stability and oxygen evolution, including oxygen-evolving enhancer proteins (OEE1 and OEE2) and a PsbP-domain protein (Figure 5 and Table 1). Because OEE/PsbP components stabilize the oxygen-evolving complex and thylakoid function, their preservation offers a direct molecular explanation for the higher ΦPSII observed in Si-treated plants under drought.

Si also sustained Rubisco activase (RCA) isoforms under drought (Figure 5 and Table 1), which is significant because RCA is a major control point for maintaining Rubisco activation and CO_2_ assimilation under stress. Drought-associated RCA decline is widely reported and is often linked to reduced carbon fixation and growth; therefore, RCA maintenance in Si-treated soybean suggests an additional route by which Si can sustain photosynthetic capacity even when CO_2_ supply is limited by stomatal closure. Together, preservation of OEE/PsbP and RCA supports a model in which Si buffers drought-induced non-stomatal limitations by stabilizing both thylakoid energy conversion and stromal carbon assimilation capacity.

### 3.4. Chloroplast Stress Protection and Nitrogen Assimilation

Beyond core photosynthesis, Si influenced chloroplast stress protection and metabolic balance. Higher abundance of a dehydrin-like protein and the chloroplast co-chaperonin CPN20 in Si-treated plants (Figure 5 and Table 1) is consistent with enhanced proteostasis, improved protein folding, and stabilization during dehydration. In addition, Si helped maintain chloroplast glutamine synthetase abundance under drought, suggesting improved coordination of nitrogen assimilation and/or photorespiratory nitrogen recycling with photosynthetic function, which may contribute to chlorophyll retention and reduced induction of stress-associated secondary metabolites (Figure 3).

### 3.5. Mechanistic Interpretation and Study Limitations

Importantly, our data support association but do not fully resolve causality. The observed proteomic shifts could reflect a direct effect of Si on chloroplast signaling or gene expression (for example, via hormone balance, calcium-associated processes, or redox signaling), but they could also arise indirectly because Si-treated plants experienced less physiological stress (better hydration, lower ROS pressure and reduced protein damage/turnover). Distinguishing these alternatives will require targeted follow-up, such as time-course analyses prior to visible symptom divergence, parallel measurement of chloroplast ROS and protease activity, and transcript-protein comparisons for key targets (e.g., RCA, OEE proteins, chaperones, and dehydrin).

Overall, the novelty of this study lies in defining a soybean-specific, chloroplast-level signature of Si-mediated drought tolerance, centered on preservation of PSII/OEC components, Rubisco activation machinery, and stress-protective proteostasis factors. These findings provide a mechanistic framework for agronomic Si management in soybean and highlight chloroplast proteins and pathways that could be prioritized for genetic or breeding strategies aimed at improving drought resilience in a crop with intermediate Si accumulation capacity. A limitation of this work is that Si was supplied as sodium silicate; although we included a NaCl treatment to control for sodium, we did not include a potassium silicate control, so counter-ion effects cannot be fully excluded and should be addressed in future studies. This knowledge enhances our understanding of plant stress physiology and could guide future efforts to develop crops with greater resilience to climate variability.

## 4. Materials and Methods

### 4.1. Plant Materials and Growth Conditions

Soybean “Williams 82” cultivar (*Glycine max* L. cv. Williams 82/accession PI 518671) has been selected for this study due to the availability of its sequenced genome [56]. The soybean seeds were grown in plastic cups (six cups per tray) filled with sand in a growth chamber (12 h day/12 h night, 25 °C). Plants were watered with 300 mL of distilled water in each tray every other day until treatments began. At the cotyledon stage, Si and control treatments were applied by adding solutions to the trays: 2 mM sodium silicate (Na_2_SiO_3_) for Si treatment, 4 mM sodium chloride (NaCl) for the ionic control (to account for sodium effects), or continued distilled water for the water control. These treatments (Si, NaCl, or H_2_O) were applied for 14 days, after which drought stress was imposed by withholding irrigation for 7 days. Six treatment groups were established, combining Si or NaCl with either drought (D) or well-watered conditions (W): (1) Si + D, (2) Si + W, (3) NaCl + D, (4) NaCl +W, (5) H_2_O + D, and (6) H_2_O + W. A quarter-strength Hoagland nutrient solution was supplied to each tray during the experiment to provide baseline nutrition. After 7 days of drought (at the second trifoliate stage), plants from each treatment were sampled for physiological measurements and chloroplast isolation.

### 4.2. Physiological Parameter Measurements

Various physiological parameters were measured on recently fully expanded trifoliate leaves of all plants, with three biological replicates per treatment. Measurements were taken both before drought imposition and at the end of the drought period, which is 7 days without water. Stomatal conductance and transpiration rate were measured between 9:00 a.m. and 11:00 a.m. using a Li-COR 600 porometer/fluorometer (Li-COR, Lincoln, NE, USA). Photosynthetic performance was assessed as the quantum efficiency of photosystem II (PhiPS2) using the fluorometer function. Leaf chlorophyll content, flavonoid content, and anthocyanin content were measured non-destructively using a Dualex Scientific™ leaf clip sensor (Force-A, Orsay, France) on the same leaves [57].

### 4.3. Chloroplast Isolation

Chloroplasts were isolated and purified by Percoll gradient centrifugation as described by Wijk et al. [58] with modifications. Soybean leaves were collected (approximately 10 g) and ground in a Warren blender containing 100 mL of grinding buffer (50 mM HEPES-KOH (pH 8.0), 330 mM sorbitol, 2 mM tetrasodium EDTA, 5 mM ascorbic acid, 5 mM cysteine, 0.05% BSA) at 4 °C for 3 times (10 s each) at setting 4. The resulting homogenate was filtered through two layers of Miracloth into a 250-mL centrifuge tube on ice. Then, the two balanced centrifuge tubes with the filtered homogenate were placed into opposite positions within the SLA-1500 rotor of the centrifuge. They were then centrifuged at 200× *g* for 5 min at 4 °C. Taking care not to disturb the pellets at the bottom of the tube, the supernatant (containing most of the chloroplasts and being turbid and deep green) was poured into a clean pre-chilled centrifuge tube. Then, the chloroplast suspension was centrifuged at 2000× *g* for 5 min at 4 °C. We poured off most of the supernatant and resuspended the chloroplast pellet in the residual about 500 µL supernatant by rotating the tube on ice. Load the resuspended chloroplasts onto the top of the Percoll step gradient (40%–85%) using a 1-mL pipette tip. Pipetted very slowly without disturbing the gradient and centrifuged the loaded Percoll gradient in a swing-out rotor at 3750× *g* for 20 min at 4 °C. After centrifugation, remove the tube carefully and place it on ice. The lower green band at the 40%–85% interface contains intact chloroplasts, whereas the upper band contains broken chloroplasts. Broken chloroplasts were removed and discarded by pipetting, and then the intact chloroplasts were removed using a 1-mL pipette tip and transferred into a pre-cooled 30 mL Nalgene tube. Add 25 mL wash buffer (50 mM HEPES-KOH (pH 8.0), 330 mM sorbitol, 2 mM tetrasodium EDTA) to the chloroplasts and invert the tube 3 times to wash off the Percoll. And then centrifuge the chloroplasts in a swing-out rotor at 1000× *g* for 5 min at 4 °C. Gently pour off the supernatant and resuspend the chloroplasts in 150 µL fresh wash buffer. The purified chloroplasts were stored at −20 °C for further proteomic analysis.

### 4.4. Protein Extraction from Chloroplast

Proteins were extracted from isolated chloroplasts using the TCA (trichloroacetic acid)-acetone method by Mechin et al. [59] with minor modifications. The isolated chloroplasts stored in 2 mL tubes at −20 °C were frozen in liquid nitrogen, and then the chloroplast sample was crushed with a blue pestle. Then add 2 mL chilled 10% TCA/acetone containing 0.07% 2-mercaptoethanol into this mixture to dissolve the pellet completely by vortexing and incubated at −20 °C for 1 h. After incubation, centrifuge at 4 °C for 10 min at 10,000× *g*. After centrifuging, the supernatant was removed by pipetting without touching the pellet. The pellet was resuspended in 2 mL of cold acetone containing 0.07% 2-mercaptoethanol and incubated at −20 °C for 1 h. After 1-h incubation, the proteins were centrifuged at 4 °C for 15 min at 10,000× *g*, and the supernatant was discarded. Repeat the rinsing step three times to get a white pellet. Dry the pellet under vacuum for about 10 min to fully eliminate acetone in the SpeedVac. The dried protein powder was then stored at −80 °C for further analysis.

### 4.5. Two-Dimensional Gel Analysis of Chloroplast Proteins

Chloroplast protein powder was resuspended in rehydration buffer following the method of Sah et al. [60]. The protein content in supernatant was quantified using the modified Bradford assay [61]. Proteins were separated using two-dimensional gel electrophoresis (2-DE). Isoelectric focusing (IEF) was performed on immobilized pH gradient strips with a pH range of 4–7 (Bio-Rad Laboratories, Hercules, CA, USA). Following IEF, strips were equilibrated and loaded onto sodium dodecyl sulfate-polyacrylamide gel electrophoresis (SDS-PAGE) gels for the second dimension as described by Sah et al. [60]. The gels were stained with colloidal Coomassie solution to visualize proteins resolved by two-dimensional gel electrophoresis. After gel staining, the 2-DE images were captured using a ChemiDoc Touch Imaging System (Bio-Rad Laboratories). Protein spot intensities were detected and analyzed with ImageJ software (https://imagej.net/ij/) as described by Natale et al. [62].

### 4.6. Protein Digestion and Mass Spectrometry Analysis

Protein spots on stained 2-DE gels were cut and digested with trypsin as described by Sah et al. [60]. Mass spectrometry (MS) analysis and database search were conducted at the Vermont Genetics Network Proteomics Facility of the University of Vermont, as described below. Tryptic digests were analyzed on the Thermo Q-Exactive Plus mass spectrometer coupled to an EASY-nLC 1200 system (Thermo Fisher, Waltham, MA, USA). Peptides were separated on a fused silica capillary (15 cm × 100 µm I.D) packed with Halo C18 (2.7 µm particle size, 90 nm pore size, Michrom Bioresources, Auburn, CA, USA) at a flow rate of 300 nL/min. Peptides were loaded into the mass spectrometer via a nanospray ionization source at a spray voltage of 2.2 kV. Mass spectrometry data were acquired in a data-dependent top 10 mode, and the lock mass function was activated (m/z, 371.1012; use lock masses, best; lock mass injection, full MS). Full scans were acquired from m/z 350 to 1700 at 70,000 resolution (automatic gain control [AGC] target, 1e6; maximum ion time [max IT], 100 ms; profile mode). Resolution for dd-MS^2^ spectra was set to 17,500 (AGC target: 1x10^6^) with a maximum ion injection time of 50 ms. The normalized collision energy was 27 eV. A gradient of 0 to 35% acetonitrile (0.1% formic acid) over 120 min was applied. The spectra were searched against the Soybean protein database: Williams 82 Assembly 4 Annotation 1 (Wm82.a4.v1) Protein Sequences (https://legacy.soybase.org/GlycineBlastPages/blast_descriptions.php (accessed on 30 May 2024) by Proteome Discoverer 2.4 (Thermo Fisher Scientific, Waltham, MA, USA). The search parameters permitted a 10-ppm precursor MS tolerance and a 0.02 Da MS/MS tolerance. Carboxymethylation of cysteines was set up as a fixed modification, and oxidation of methionine (M) as a dynamic modification. Up to two missed tryptic cleavages of peptides were considered, with the false-discovery rate set to 1% at the peptide level.

### 4.7. Gene Ontology Enrichment and Pathway Analysis

Identified chloroplast proteins were functionally annotated using gene ontology (GO) and pathway analysis. GO enrichment analysis of the identified differential proteins was conducted using agriGO v2.0 [63], with default parameter settings. The soybean genome (Wm82.a4.v1) was used as the background or reference for the analysis. Each protein was classified into biological process (BP), molecular function (MF), and cellular component (CC) categories, and overrepresentation of GO terms was assessed against the soybean genome background. For pathway enrichment analysis, the genes encoding the identified differential proteins were analyzed with KEGG (Kyoto Encyclopedia of Genes and Genomes) annotation data for the soybean genome (Wm82.a4.v1) with TBtools, a software suite for biological data analysis [64]. A *p*-value threshold of <0.05 was used to evaluate the significant enrichments in GO terms and KEGG pathways.

### 4.8. Statistical Analysis

Physiological data (e.g., stomatal conductance, chlorophyll content) were analyzed by one-way ANOVA followed by Tukey’s HSD test at *p* < 0.05 to determine significant differences between treatments. Statistical analysis was performed in R v4.2.0 (R Foundation for Statistical Computing, Vienna, Austria) using the doebioresearch package for ANOVA and post-hoc comparisons. Protein expression differences were assessed qualitatively based on spot intensities in Coomassie-stained gels and confirmed by the presence/absence in MS identification. All experiments were conducted with at least three biological replicates.

## 5. Conclusions

This study investigated how soybean plants respond to drought stress, with a focus on the mitigating effects of Si supplementation. We analyzed both physiological traits and changes in chloroplast proteins. Under well-watered conditions, silicon supplementation had minimal impact on basic physiological parameters. However, during drought stress, it provided significant benefits. While Si did not significantly affect stomatal conductance or transpiration under drought conditions, it did noticeably enhance the quantum efficiency of PSII and help preserve chlorophyll content in drought-stressed plants. The observed positive correlation between chlorophyll retention and sustained light-dependent reaction efficiency suggests that Si played a crucial role in maintaining photosynthetic function during drought. At the proteomic level, we identified notable differences between Si-treated plants and controls under drought stress. A total of 13 chloroplast proteins were differentially expressed in response to Si supplementation. These proteins are involved in various biological processes, particularly photosynthesis (e.g., Rubisco activase, oxygen-evolving complex proteins) and stress responses (e.g., dehydrin and chaperonin). Silicon supplementation increased the abundance of proteins involved in carbohydrate and energy metabolism, such as Rubisco activases, while preventing drought-induced decline of these proteins. Additionally, Si maintained or increased levels of protective proteins, including oxygen-evolving enhancer proteins, PsbP-domain proteins, glutamine synthetase, dehydrin, and 20 kDa chaperonin under drought conditions. Our findings suggest that Si enhances soybean plants’ drought tolerance by sustaining core photosynthetic processes and stress response mechanisms at the chloroplast level. It appears to improve the functioning of proteins involved in carbon assimilation (Rubisco activase) and the photosynthetic electron transport chain (oxygen-evolving complex components), thereby sustaining continued photosynthesis during drought. Silicon also helped preserve nitrogen assimilation capacity (glutamine synthetase activity), which may contribute to higher chlorophyll content. Proteins that respond to dehydration stress, such as dehydrin and the 20 kDa chaperonin, showed improved expression with Si supplementation, indicating an enhanced protective capacity. It is noteworthy that most dicot plants, including soybeans, accumulate only small amounts of Si due to limited expression of Si transporter genes. This suggests that enhancing Si uptake could significantly benefit these plants. Genetic engineering or selective breeding to increase Si uptake and accumulation in soybeans could amplify the natural protective effects observed in this study. The molecular and proteomic insights provided here can guide these efforts. By understanding which proteins and pathways are influenced by Si, we can target modifications through breeding or biotechnology to develop more drought-tolerant soybean varieties. Leveraging Si’s benefits, either through soil amendments or genetic improvements, holds promise for maintaining crop productivity amid increasing drought stress driven by climate change.

## Figures and Tables

**Table 1:** Differentially expressed chloroplast proteins from soybean plants in response to Si supplementation.

Spot No ^a^	Protein Name	Accession No ^b^ (UniProt ID)	Gene Locus	Theoretical pI/MW ^c^	Experimental pI/MW ^d^	Change Pattern ^e^
1	Rubisco activase, chloroplastic isoform C	D4N5G3 (D4N5G3_SOYBN)	Glyma.11G221000	6.27/48,665	5.70/48,100	↑ (Si, Si + D)
2	Rubisco activase, chloroplastic isoform C	D4N5G3 (D4N5G3_SOYBN)	Glyma.11G221000	6.27/48,665	5.90/48,100	↑ (Si, Si + D)
3	Rubisco activase, chloroplastic isoform A	C6T859 (C6T859_SOYBN)	Glyma.18G036400	6.79/48,624	6.10/48,400	↑ (Si, Si + D)
4	Rubisco activase, chloroplastic isoform A	C6T859 (C6T859_SOYBN)	Glyma.18G036400	6.79/48,624	6.30/48,400	↑ (Si, Si + D)
5	Glutamine synthetase, chloroplastic	I1MFC4 (I1MFC4_SOYBN)	Glyma.15G102000	6.73/47,692	6.60/47,900	↑ (Si + D)
6	Oxygen-evolving enhancer protein 1, chloroplastic	C6T7N2 (C6T7N2_SOYBN)	Glyma.02G061100	6.32/34,628	6.20/34,100	↑ (Si + D)
7	33 kDa subunit of oxygen evolving system of photosystem II, chloroplastic	C6TC92 (C6T7N2_SOYBN)	Glyma.11G061300	5.58/34,778	6.20/34,100	↑ (Si + D)
8	Splicing factor 3b, subunit 4, chloroplastic	I1JQ98 (I1JQ98_SOYBN)	Glyma.03G202900	5.23/34,304	5.10/33,900	↑ (Si + D)
9	RNA-binding protein, chloroplastic	A0A0R0JQU1 (A0A0R0JQU1_SOYBN)	Glyma.05G043200	4.85/32,914	4.70/32,100	↑ (Si)
10	Oxygen-evolving enhancer protein 2, chloroplastic	I1M712 (I1M712_SOYBN)	Glyma.14G031800	7.69/28,597	6.40/27,900	↑ (Si + D)
11	Oxygen-evolving enhancer protein 2-1, chloroplastic	I1KXW9 (I1KXW9_SOYBN)	Glyma.08G304200	6.96/28,230	6.30/27,800	↑ (Si + D)
12	20 kDa chaperonin, chloroplastic	C6TJG0 (C6TJG0_SOYBN)	Glyma.09G072100	6.77/26,656	6.10/27,100	↑ (Si + D)
13	PsbP domain-containing protein 6, chloroplastic	I1N8N4 (I1N8N4_SOYBN)	Glyma.19G126700	5.97/28,417	5.80/27,700	↑ (Si + D)
14	Dehydrin-like protein	Q39805 (Q39805_SOYBN)	Glyma.09G185500	6.58/23,718	6.80/28,300	↑ (Si + D)
15	ATP synthase subunit beta, chloroplastic	A0A898CT38 (A0A898CT38_SOYBN)	atpB	5.40/53,755	6.30/54,100	↓ (Si + D)

^a^. Spot Nos correspond to the numbers on the gels in Figure 5. ^b^. Accession Nos are the accession numbers in the UniProt database (http://www.uniprot.org/). ^c^. Theoretical isoelectric point (pI) and molecular weight (MW) of proteins were calculated from their sequences in the UniProt database. ^d^. Experimental pI and MW values of proteins were determined by their respective positions along the two distinct dimensions of 2D gel electrophoresis separation. ^e^. “↑” or “↓” indicates the protein level increased or decreased in response to Si supplementation under control and drought (D) conditions.

**Table 2:** Functional classification of differentially expressed chloroplast proteins from soybean plants in response to Si supplementation.

Function	Protein Name	Gene Locus	References
**Photosynthesi** **s**	Rubisco activase, chloroplastic isoform A	Glyma.18G036400	Yin et al. [37]; Rollins et al. [38]
	Rubisco activase, chloroplastic isoform C	Glyma.11G221000	Maruyama et al. [39]; Da Silva et al. [40]
	Oxygen-evolving enhancer protein 1, chloroplastic	Glyma.02G061100	Hajheidari et al. [41]
	Oxygen-evolving enhancer protein 2, chloroplastic	Glyma.14G031800	Zadraznik et al. [30]
	Oxygen-evolving enhancer protein 2-1, chloroplastic	Glyma.08G304200	Katam et al. [42]
	33 kDa subunit of oxygen evolving system of photosystem II	Glyma.11G061300	Yuan and Cline [43]
	PsbP domain-containing protein 6, chloroplastic	Glyma.19G126700	Tamburino et al. [29]
**Metabolism**	Glutamine synthetase, chloroplastic	Glyma.15G102000	Du et al. [44]
	ATP synthase subunit beta, chloroplastic	atpB	Wang et al. [45]; Kim et al. [46]
**RNA processing and stability**	Splicing factor 3b, subunit 4, chloroplastic	Glyma.03G202900	Griffin [47]
	RNA-binding protein, chloroplastic	Glyma.05G043200	Ambrosone et al. [33]
**Protein folding**	20 kDa chaperonin, chloroplastic	Glyma.09G072100	Nazari et al. [48]
**Stress response**	Dehydrin-like protein	Glyma.09G185500	Szlachtowska and Rurek [34]

## Data Availability

The mass spectrometry proteomics data have been deposited to the ProteomeXchange Consortium via MassIVE (https://massive.ucsd.edu/) with the dataset identifier MSV000092721.

## Supplementary Materials

The following supporting information can be downloaded at: https://www.mdpi.com/article/10.3390/plants15030497/s1, Table S1. Integrated intensity data obtained from ImageJ-based analysis for the protein spots shown in Figure 5. ND stands for Not Detected (the spot is absent in the gels).
