# Supplementary material for: Silicon Modulates the Chloroplast Proteome to Enhance Drought Tolerance in Soybean
Source: Plants (Basel). 2026 Feb 5;15(3):497. doi: 10.3390/plants15030497 (PMC12898996; doi:10.3390/plants15030497)
Supplement: Supplementary file 1 [file plants-15-00497-s001.zip › plants-4108799-supplementary.pdf]

Supplementary Table S1. Integrated intensity data obtained from ImageJ-based analysis for the protein spots shown in Figure 5. ND stands for Not Detected (the spot is absent in the gels).

Spot Number	H_2O + Drought	NaCl + Drought	Na_2SiO_3 + Drought	H_2O + Control	NaCl + Control	Na_2SiO_3 + Control
1	0.528	0.565	1.839	0.791	0.910	1.932
2	0.802	0.970	2.396	0.858	0.745	2.702
3	0.623	0.638	1.223	0.869	0.805	1.728
4	0.630	0.731	1.111	0.879	0.644	1.219
5	0.297	0.330	0.611	0.690	0.642	0.748
6	2.609	3.512	5.522	1.832	2.934	2.354
7	1.875	2.184	3.775	0.809	1.565	1.457
8	ND	ND	1.606	ND	ND	ND
9	ND	ND	ND	ND	ND	0.575
10	1.551	1.902	4.144	1.194	2.156	2.742
11	1.411	1.927	3.440	1.490	2.605	2.126
12	0.606	0.721	1.557	0.642	0.970	0.917
13	1.077	1.646	3.274	1.129	2.066	1.961
14	0.263	0.277	0.810	0.511	0.231	0.347
15	2.525	1.849	0.715	1.004	3.523	2.114
